# The correlation between plasma brain-derived neurotrophic factor and cognitive function in bipolar disorder is modulated by the BDNF Val66Met polymorphism

**DOI:** 10.1038/srep37950

**Published:** 2016-12-01

**Authors:** Sheng-Yu Lee, Tzu-Yun Wang, Shiou-Lan Chen, Yun-Hsuan Chang, Po-See Chen, San-Yuan Huang, Nian-Sheng Tzeng, Liang-Jen Wang, I. Hui Lee, Kao Chin Chen, Yen Kuang Yang, Yi-Hsin Yang, Ru-Band Lu, Cheng-Sheng Chen

**Affiliations:** 1 Graduate Institute of Medicine, College of Medicine, Kaohsiung Medical University, Kaohsiung, Taiwan; 2 Department of Psychiatry, Kaohsiung Veterans General Hospital, Kaohsiung, Taiwan; 3 Department of Psychiatry, College of Medicine, National Cheng Kung University, Tainan, Taiwan; 4 Lipid Science and Aging Research Center, College of Medicine, Kaohsiung Medical University, Kaohsiung, Taiwan; 5 Deprtment of Psychology, Asia University, Taichung, Taiwan; 6 Addiction Research Center, College of Medicine, National Cheng Kung University, Tainan, Taiwan; 7 Department of Psychiatry, Tri-Service General Hospital, National Defense Medical Center, Taipei, Taiwan; 8 Student Counseling Center, National Defense Medical Center, Taipei, Taiwan; 9 Department of Child and Adolescent Psychiatry, Kaohsiung Chang Gung Memorial Hospital and Chang Gung University College of Medicine, Kaohsiung, Taiwan; 10 Department of Psychiatry, National Cheng Kung University Hospital, Dou-Liou Branch, Yunlin, Taiwan; 11 Institute of Behavioral Medicine, College of Medicine, National Cheng Kung University, Tainan, Taiwan, National Cheng Kung University, Tainan, Taiwan; 12 School of Pharmacy, Kaohsiung Medical University, Kaohsiung, Taiwan; 13 Center for Neuropsychiatric Research, National Health Research Institutes, Miaoli, Taiwan; 14 Department of Psychiatry, Kaohsiung Medical University Hospital, Kaohsiung Medical University, Kaohsiung, Taiwan; 15 Department of Psychiatry, Faculty of Medicine, College of Medicine, Kaohsiung Medical University Kaohsiung, Taiwan

## Abstract

We explored the effect of the Brain-derived neurotrophic factor (*BDNF*) Val66Met polymorphism (rs6265) on correlation between changes in plasma BDNF levels with cognitive function and quality of life (QoL) after 12 weeks of treatment in bipolar disorder (BD). Symptom severity and plasma BDNF levels were assessed upon recruitment and during weeks 1, 2, 4, 8 and 12. QoL, the Wisconsin Card Sorting Test (WCST), and the Conners’ Continuous Performance Test (CPT) were assessed at baseline and endpoint. The *BDNF* Val66Met polymorphism was genotyped. Changes in cognitive function and QoL over 12 weeks were reduced using factor analysis for the evaluation of their correlations with changes in plasma BDNF. Five hundred forty-one BD patients were recruited and 65.6% of them completed the 12-week follow-up. Changes in plasma BDNF levels with factor 1 (WCST) were significantly negatively correlated (*r* = −0.25, *p* = 0.00037). After stratification of BD subtypes and *BDNF* genotypes, this correlation was significant only in BP-I and the Val/Met genotype (*r* = −0.54, *p* = 0.008). We concluded that changes in plasma BDNF levels significantly correlated with changes in WCST scores in BD and is moderated by the *BDNF* Val66Met polymorphism and the subtype of BD.

Bipolar disorder (BD) is characterized by recurrent episodes of dysregulated moods^1^,^2^. With high heritability^3^, genetic factors had been regarded as an important etiology for BD. The most frequently seen subtypes of BD are bipolar I disorder (BP-I) and bipolar II disorder (BP-II). Being a chronic mental disorder, BD is increasingly regarded as a neurodegenerative disorder supported by imaging studies^4^,^5^. Brain-derived neurotrophic factor (BDNF) is an important protein for neuron development, growth and survival^6^. BDNF is robustly expressed in the brain area regulating memory and emotion: the cortex and hippocampus^7^. Therefore, BDNF may play an important role in the pathogenesis of BD^4^,^8^. A meta-analysis reported that BDNF levels are lower during manic and depressive episodes, but that, after treatment for acute mania, they are not significantly different from controls^9^. Some studies^10^,^11^,^12^ reported significantly lower serum BDNF levels in patients with BD suffering from manic or depressive episodes. Serum BDNF levels increased after patients received treatment with antidepressants and mood stabilizers^13^,^14^. However, BDNF levels still decrease with age and duration of illness in euthymic BD patients^9^. With above findings, peripheral BDNF has been proposed as a candidate biomarker reflecting mood severity and disease progression for BD^9^. *In vivo* study^15^ demonstrated that the brain and plasma BDNF levels went through similar variation during maturation and aging. In this way, peripheral BDNF levels might echo BDNF levels in the brain^16^,^17^. In addition, Laske *et al*.^18^ suggested that in Alzheimer’s disease, decrease in plasma BDNF levels may reflect the degree of neuronal degeneration. Hence, it is suggested that plasma BDNF levels might also suggest the degree of neuronal degeneration in BD^11^.

The *BDNF* gene is located on chromosome 11p13 in human. One functional single-nucleotide polymorphism, the *BDNF* Val66Met (rs6265) polymorphism, causing substitution from valine (Val) to methionine (Met) at codon 66^19^,^20^ and resulting into ineffective BDNF trafficking and decreased secretion of activity-dependent BDNF^19^. Over-transmission of the Val allele has been associated with risk of BD in North American and European studies^21^,^22^ but not supported by studies on Asian populations^23^,^24^. In addition, the *BDNF* gene has been linked to response to antidepressants^25^, mood stabilizers^26^, and treatment outcome of depressive symptoms^27^ in patients with BD.

Even after the remission of symptoms, cognitive function damage remains in BD^28^. Martinez-Aran *et al*.^29^ reported that even in euthymic states, impaired executive function and verbal memory abilities were found in patients with BD than those of healthy controls. Some researchers^30^ have suggested that cognitive impairment in euthymic patients with BD might be associated with the underlying pathophysiology of BD, such as neurodegeneration and a decrease in BDNF expression levels. Thus, the *BDNF* Val66Met polymorphism, which modulate BDNF secretion, might be associated with cognitive impairment in BD^31^. Moreover, it was reported that having the Met allele and lower BDNF expression levels might affect hippocampal structure and function and impair cognition processes^32^.

Quality of life (QoL), is defined by the World Health Organization (WHO) as “a state of physical, mental and social wellbeing, according to one’s self-perceptions of their position in life, and in relation to their goals, expectations, standards and concerns”^33^. This WHOQOL consists of 4 aspects: physical health, psychological functioning, social relationships, and environment^34^. Impairments in QoL have been found in BD^35^, and QoL has been proposed to be a more comprehensive measure of treatment response beyond clinical symptoms^36^. The *BDNF* Val66Met polymorphism has been associated with improvement in QoL scores in patients with major depressive disorder and treated with fluoxetine^37^. However, the association of the *BDNF* Val66Met polymorphism and QoL in BD patients has never been examined.

Most studies have focused on the cross-sectional association, not the longitudinal association of changes in cognitive function and QoL with change of plasma BDNF levels or the influence of the *BDNF* Val66Met polymorphism. By analyzing cross-sectional data, only inter-subject correlation was formed. However, from longitudinal design, we will be able to detect the correlation of changes in plasma BDNF and changes in cognitive function and QoL from within-individual perspective. We believe such approach may better reflect the real-life change and correlation of plasma BDNF levels, cognitive function and QoL, while the modulation effect of the *BDNF* Val66Met polymorphism may also be assessed. Therefore, the aims of this study were to investigate the correlation of longitudinal changes in plasma BDNF levels with longitudinal changes of cognitive function and QoL stratified by the *BDNF* Val66Met polymorphism in a 12-week follow-up study in patients with BD.

## Methods

### Patient Selection

The Institutional Review Board for the Protection of Human Subjects at National Cheng Kung University Hospital and Tri-Service General Hospital examined and approved the research protocol. The methods were carried out in accordance with the approved guidelines. All participants signed written informed consent forms after the study had been well explained for.

This study is a secondary analysis of the combination of 2 clinical trials. The original studies were (1) a randomized, double-blind, controlled 12-week trial that investigated the add-on effect of memantine on BP-II treated using valproate (VPA)(Trial registration: NCT01188148)^38^ and (2) a randomized, double-blind, controlled 12-week trial investigating the add-on effect of dextromethorphan (DM) in BD treated by valproate (VPA) (Trial registration: NCT01188265)^39^. We analyzed all the BD patients from the two studies but not the healthy controls.

Patients with BP-I and BP-II were recruited from outpatient and inpatient settings. All participants were first interviewed by senior psychiatrists according to the diagnostic criteria of Diagnostic and Statistical Manual of Mental Disorders fourth edition (DSM-IV). The patients then received a structural interview conducted by clinical psychologist using the Chinese Version of the Modified Schedule of Affective Disorder and Schizophrenia-Life Time (SADS-L)^40^, which has good inter-rater reliability^41^. Patients diagnosed with other major mental illnesses, borderline personality disorder, substance abuse or dependence, and cognitive disorders other than BP-I or BP-II were excluded.

The diagnostic criteria for hypomania in the current study utilized a 2-day minimum duration instead of the 4-day duration defined by the DSM-IV-TR (Text Revision)^42^ criteria. We chose the 2-day duration for hypomanic since such definition supported by community samples^43^,^44^,^45^,^46^,^47^,^48^.

### Study Design

The patients received add-on treatment of either DM (30 mg/day or 60 mg/day), memantine (5 mg/day), or placebo for 12 weeks after a baseline assessment augmenting their open-label VPA treatment (500 mg and 1000 mg daily [50–100 μg/ml in plasma]). Severity of mood symptom was assessed at baseline and on day 7 of weeks 1, 2, 4, 8 and 12. Less than 8 mg/day of concomitant lorazepam was permitted for insomnia or anxiety during the study. Fluoxetine (20 mg/daily) and Risperidone (1–6 mg/daily) and were allowed during depressive and manic stages, respectively.

The 17-item Hamilton Depression Rating Scale (HDRS)^49^,^50^ was used to assess the severity of depressive symptoms; the 11-item Young Mania Rating Scale (YMRS) was used to assess the severity of manic symptoms^51^,^52^. Patients need to score HDRS ≥ 18 to be recruited.

We used Wisconsin Card Sorting Test (WCST) and the Conners’ Continuous Performance Test (CPT) to assess cognitive function. The WCST is regarded as a performance test for frontal lobe dysfunction^53^ by measuring one’s ability to perform executive functions in the following categories: Categorization, abstraction reasoning, maintaining sets, set switching, strategic planning, and modulating impulsive responding. Performance on the WCST was scored in terms of the total number of errors (TNE), perseverative errors (PE), perseverative response (PR), number of categories completed (NCC), and trials to complete the first category (TCC).

The CPT consists of a set of performance measures that include the number of errors of omission and errors of commission, hit reaction time (HRT), HRT standard error (HRT SE), variability and detectability (d’). Errors of omission stands for ones failing to respond to the target stimulus. Errors of commission stands for one responding to a non-target stimulus. HRT shows the mean response time (milliseconds) for all target stimulus over the full six trial blocks. HRT SE means the consistency of response times and expresses the SE response to targets. Detectability (d’) stands for how well the examinee discriminates between targets and non-targets.

The Chinese version of the brief version of the World Health Organization Quality of Life instrument (WHOQOL-BREF)^54^, which is widely used in Taiwan^55^, was implemented to access the quality of life in BD patients at baseline and endpoint. The WHOQOL-BREF is consisted 28 items dividing into four domains: physical, psychological, social relationships and environmental. The score of each domain ranged from 4 to 20, computed by multiplying the average scores of all items by 4. Higher scores implies a better quality of life.

### Blood Samples and Genotyping

At baseline, 10 milliliters of venous blood were collected from each participants. DNA was extracted from the lymphocytes of the blood sample. The *BDNF* Val66Met polymorphism was genotyped using a modified protocol^22^. All samples were double-checked to keep genotype error less than 5%.

Blood samples were collected between 9 am and 11 am after 8 hours of fasting at baseline and endpoint to measure Plasma BDNF level. Blood sample was drawn into a vacuum tube containing ethylenediamine tetraacetic acid (EDTA) (Greiner Bio-One Vacuette; Santa Cruz Biotechnology, Santa Cruz, CA) then kept on ice for up to 30 minutes. At 4 °C, the whole blood was centrifuged at 3000 *g* for 15 minutes to isolate plasma; then stored at −80 °C. A BDNF kit (Quantikine Human BDNF kit; R&D Systems, Minneapolis, MN), and an enzyme-linked immunosorbent assay (ELISA) reader (SpectraMax-M2; Molecular Devices, Sunnyvale, CA) with minimum detectable dose of 80 pg/ml were used to assess the level of plasma BDNF. All samples were analyzed twice.

### Statistical Analysis

The demographic, clinical characteristics, performance in WCST and CPT, and WHOQOL of the patients at baseline and endpoint including the HDRS and YMRS scores and plasma BDNF levels were compared with paired-t test. Performance in WCST and CPT was reported as standardized score (T-score).

The change of scores of the WCST, CPT, and WHOQOL measures over 12 weeks were reduced by a principal components analysis. Factors yielding eigenvalues greater than 1.00 were retained for varimax rotation with Kaiser Normalization. Change of plasma BDNF levels underwent arithmetic transformations using log (*x*)+ 1 to yield approximately normal distributions for further statistical analysis. We then investigated the correlation between each composite scores with transformed changes of plasma BDNF levels using Pearson’s correlation and further stratified by subtypes of BD (BP-I and BP-II) and the *BDNF* genotypes.

The current study used SPSS 18 for Windows (Chicago, SPSS Inc.) for statistical computations. P < 0.05 was set as significant.

## Results

We recruited 541 patients with BD. Three hundred fifty-five (65.6%) of the 541 patients completed the 12-week follow-up and 186 (34.4%) dropped out. The detailed reasons for discontinuing are given elsewhere (Chen *et al*.^39^; Lee *et al*.^38^).

There was a significant attenuation of clinical severity (HDRS and YMRS) and improvement in several subscores of the WCST, CPT, and WHOQOL after 12 weeks of treatment (Table 1).

Factor analysis was used to condense the change of the WCST, CPT, and QoL measures to reduce type I error. Four factors from the varimax rotation were retained; each yields eigenvalues greater than 1.00: Factor 1 is a composite score for WCST, factor 2 for CPT impulsivity, factor 3 for CPT distractibility, and factor 4 for WHOQOL. The four factors, which had eigenvalues of 3.78, 3.04, 2.03, and 1.42, accounted for 73.4% of the total matrix variance. The factor loading of each item are listed in Table 2.

**Figure 1:**
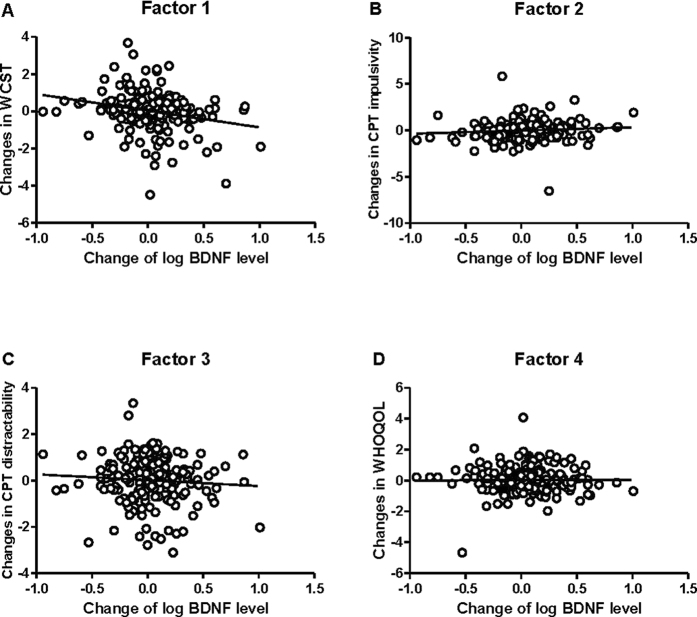
Scatter plot between change in log (plasma BDNF level) and factor scores of changes in (**A**) WCST, (**B**) CPT impulsivity, (**C**) CPT distractibility, and (**D**) WHOQOL.

Correlation between changes of plasma BDNF and the 4 composite factors obtained from factor analysis representing changes in cognitive functions and WHOQOL over 12 weeks (Fig. 1) showed a significant negative correlation between plasma BDNF levels with factor 1 (WCST) (*r* = −0.25, p = 0.00037) (Table 3). A decrease of factor 1 (WCST) indicates an improvement in executive functions. After the patients had been stratified by their BD subtypes (BP-I and BP-II) and their *BDNF* Val66Met polymorphism genotypes (Val/Val, Val/Met, and Met/Met), the correlation between factor 1 (WCST) and the change of plasma BDNF level was only in patients with BP-I and the *BDNF* Val66Met Val/Met genotype (*r* = −0.54, p = 0.008) (Table 3). To correct for multiple comparison in a more conservative way, we set significant level at p = 0.05/24 = 0.002. However, the correlation between change of BDNF and change of WCST in patients with BP-I and the *BDNF* Val66Met Val/Met genotype did not survive such stringent correction for multiple comparison.

## Discussion

We found a significant correlation between improvements in WCST scores and increases in plasma BDNF levels in all subtypes of BD. After the analysis had been stratified by BDNF Val66Met genotypes and the subtypes of BD, there was a significant correlation only between the Val/Met genotypes and BP-I, although such correlation did not survive correction for multiple comparison. This is preliminary evidence that changes of plasma BDNF affects changes in executive function in patients with BD, specifically in BP-I with a specific BDNF genotype.

Others have examined the association between plasma BDNF or BDNF polymorphisms and cognitive function. However, no other study has done a longitudinal investigation of the correlation between changes of plasma BDNF and changes of cognitive function in the subtypes of BD while considering the effects of the *BDNF* Val66Met polymorphism. Inconsistent results were found regarding the association between the *BDNF* Val66Met polymorphism and cognitive function in BD; one suggested a non-significant association^56^, one said that those with Val/Val genotypes performed better^31^, two said that those with the Met allele performed worse^57^,^58^, and two attribute the worse performance of BD patients with the Met allele to the smaller hippocampus volumes of patients with the Val/Val genotype^32^,^58^.

Some researchers have reported a non-significant correlation between peripheral BDNF levels and WCST scores^59^ and executive function in BP-I^60^,^61^. However, Dias did find that serum BDNF levels were significantly associated with verbal fluency. The correlation between BDNF and cognitive function has never been reported. Animal studies^62^ suggest that upregulating BDNF expression in the brain might improve cognitive functions. In the present study, we found a correlation between changes in peripheral BDNF levels and changes in WCST scores using a large sample size and a longitudinal study design. Our findings support the hypothesis that BDNF is a biomarker for cognitive function impairments in BD patients.

Those carrying the Val/Met genotype seems to have moderate amount of secretion of BDNF compared to the Val/Val genotype (higher BDNF) and the Met/Met genotype (lower BDNF). Govindarajan *et al*.^63^ reported that over or under amount of BDNF might have a detrimental effect on mood and behavior, and that a moderate amount of BDNF might have a mood-stabilizing effect. It was reported that BD patients with the Val/Met genotype, yielding moderate amount of BDNF, responded better to antidepressants than did carriers of the Val/Val or Met/Met genotypes^64^,^65^. In addition, the stratified analysis showed no correlation between BDNF levels and WCST scores in patients with BP-II. We previously^66^ found a trend of lower plasma levels in BP-I than in BP-II, which suggests that BDNF has a stronger effect on BP-I than on BP-II. Therefore, we hypothesized that in Val/Met genotype carriers with BP-I, peripheral BDNF levels better depict cognitive performance. However, the exact mechanisms by which a “moderate” amount of changes of BDNF affects changes of cognitive function requires further study.

Because peripheral BDNF affects mood and behavior, we chose to evaluate QoL rather than clinical symptoms as a more comprehensive measure of clinical presentation. However, we found no significant correlation between changes of plasma BDNF levels and changes of WHOQOL. This WHOQOL consists of questions about four domains: physical health, psychological functioning, social relationships, and environment^34^. We found a correlation between changes of plasma BDNF and changes of psychological performance (executive function) in the current study; therefore, it is possible that changes of plasma BDNF is not strongly associated with changes of physical health, social relationships, and environment. However, to avoid type I error from multiple correction, we condensed the changes of WHOQOL to one factor for analysis. In addition, peripheral immune markers other than plasma BDNF have been associated with QoL^67^. It will be interesting to examine the association between BDNF and cytokines in each domain of the WHOQOL in a future study.

The current study has the following limitations: First, it was suggested that changes in plasma BDNF levels might reflect BDNF levels in the human brain^17^, therefore, we used plasma BDNF in the current study to represent central BDNF level while cerebrospinal fluid (CSF) may better reflects brain-specific molecular and pathological alterations^68^. Fortunately, positive correlations between changes in plasma and CSF BDNF levels have been reported in depression^69^ and first-episode psychosis^70^. Second, peripheral BDNF levels might be affected by tobacco use^71^. However, we did not collect information about the participants’ smoking habits. Third, the diagnostic criteria for BD-II in the current study is is not widely accepted which might not be generalizable to patients diagnosed with BD-II according to the DSM-IV-TR criterion. Fourth, all patients recruited were drug naïve; our findings might generalizable to patients who have been administered antipsychotics, antidepressants, or mood stabilizers. Fifth, we focused on the changes in plasma BDNF levels and changes of cognitive parameters after a 12-week follow-up. Correlating longer-term follow-ups in our patients requires additional investigations. Finally, the significant correlation between change of BDNF and change of WCST in patients with BP-I and the *BDNF* Val66Met Val/Met genotype did not survive correction for multiple comparison. The correlation impressed is weak and should be confirmed in future study.

In conclusion, the current study provides initial evidence that increases in plasma BDNF levels were positively correlated with improvements in WCST scores regardless of the BD subtype. Such correlation is likely to remain only in those with the Val/Met genotypes and BP-I, after further stratification of the *BDNF* Val66Met genotypes and subtypes of BD. We hypothesize that because Val/Met genotype carriers have a moderate amount of BDNF secretion, peripheral BDNF levels better depict cognitive performance in BD-I with the Val/Met genotype. However, the exact mechanisms by which moderate amounts of BDNF affect cognitive function require further study; such knowledge might serve as a reference for the development of novel strategies to attenuate cognitive decline and inhibit neurodegeneration in BD.

## Additional Information

**How to cite this article**: Lee, S.-Y. *et al*. The correlation between plasma brain-derived neurotrophic factor and cognitive function in bipolar disorder is modulated by the BDNF Val66Met polymorphism. *Sci. Rep.* **6**, 37950; doi: 10.1038/srep37950 (2016).

**Publisher's note:** Springer Nature remains neutral with regard to jurisdictional claims in published maps and institutional affiliations.

## Figures and Tables

**Table 1:** Baseline and endpoint characteristics, quality of life, and cognitive function of patients with bipolar disorder.

Characteristics	Baseline	Endpoint	*Paired-t*	*p*
Number (n)	541	355		
Gender (male/female) (n)	264/277	183/172		
BDNF Val66Met polymorphism distribution (n) (%)	117/289/135 (21.6%/53.4%/25.0%)	80/183/92 (22.5%/51.5%/25.9%)	p	
Age (years) (mean (SD))	31.2 ± 11.2	30.8 ± 10.9		
Diagnosis (BP-I/BP-II) (n)	117/424	92/263		
Augmented treatment (n) (Memantine/Placebo/DM30/DM60)	115/223/102/101	81/149/67/58		
HDRS^1^ score (mean (SD))	17.9 ± 6.2	8.3 ± 5.5	22.9	3.8E-68
YMRS^2^score (mean (SD))	9.8 ± 5.0	5.3 ± 3.3	14.7	3.3E-37
Plasma BDNF level (ng/mL)	16.9 ± 10.1	17.1 ± 9.7	1.6	0.11
WHOQOL				
Physical	17.7 ± 3.9	18.6 ± 4.1	3.5	0.001
Psychological	14.5 ± 3.7	15.0 ± 3.8	2.0	0.050
Social	10.0 ± 3.0	10.6 ± 2.7	1.7	0.10
Environment	24.3 ± 5.7	25.2 ± 5.4	2.6	0.01
Wisconsin Card Sorting Test (WCST)(mean (SD))§				
Total Number of Errors (TNE)	38.9 ± 22.7	34.4 ± 22.4	4.0	0.000081
Perseverative Response (PE)	23.8 ± 20.9	20.2 ± 18.6	3.3	0.002
Perseverative Errors (PE) (mean (SD))	20.8 ± 16.2	18.1 ± 14.4	3.3	0.001
Number of Completed Categories (NCC)	5.9 ± 3.2	6.5 ± 3.4	3.6	0.00034
Trials to Complete the first Category (TCC)	21.1 ± 19.3	18.0 ± 15.7	2.2	0.03
Continuous Performance Test (CPT)§				
Omission *t*-score	61.4 ± 41.0	56.8 ± 35.3	1.3	0.03
Commission *t*-score	54.1 ± 11.5	51.6 ± 12.0	3.7	0.00023
HRT T-score	48.8 ± 12.5	48.5 ± 20.4	0.2	0.81
Hit RT Std. Error *t*-score	52.1 ± 16.0	48.9 ± 13.5	3.7	0.00024
Detectability (d′) *t*-score	52.3 ± 10.3	49.7 ± 11.6	4.1	0.000055

BP-I: bipolar I disorder; BP-II: bipolar II disorder, DM: dextromethorphan.

HDRS: Hamilton Depression Rating Scale; YMRS: Young Mania Rating Scale; BDNF: brain-derived neurotrophic factor.

^§^476 patients at baseline and 298 patients at endpoint.

**Table 2:** The structure of factors loadings produced by principal components analysis of the changes of WCST, CPT and WHOQOL measures after 12-week of treatment.

Cognitive testing and QoL	Factor 1	Factor 2	Factor 3	Factor 4
	(WCST)	(CPT impulsivity)	(CPT distractibility)	(WHOQOL)
Wisconsin Card Sorting Test (WCST)				
Total Number of Errors (TNE)	0.92	−0.00039	0.20	−0.10
Perseverative Response (PE) (mean (SD))	0.92	−0.08	0.06	0.04
Perseverative Errors (PE) (mean (SD))	0.93	−0.06	0.07	0.02
Number of Completed Categories (NCC)	−0.74	0.08	−0.26	0.11
Trials to Complete the first Category (TCC)	0.70	0.22	0.26	−0.14
Continuous Performance Test (CPT)				
Omission *t*-score	0.12	−0.03	0.68	0.24
Commission *t*-score	0.01	−0.91	−0.02	0.21
HRT *t*-score	0.25	0.44	0.80	−0.10
Hit RT Std. Error *t*-score	0.15	−0.13	0.85	0.10
Detectability (d′) *t*-score	0.08	−0.92	−0.04	0.14
WHOQOL				
Physical	−0.06	−0.09	0.08	0.83
Psychological	−0.04	−0.24	0.11	0.88
Social	−0.16	−0.48	0.11	0.69
Environment	−0.02	−0.06	0.14	0.84

*The change of scores of the WCST, CPT, and WHOQOL measures over 12 weeks were reduced by means of a principal components analysis with a set of weights for composite scores for each subjects.

*Factors yielding eigenvalues greater than 1.00 were retained for varimax rotation.

*The Rotation Method used was Varimax with Kaiser Normalization.

*The four factors, which had eigenvalues of 3.78, 3.04, 2.03, and 1.42, accounted for 73.4% of the total matrix variance.

**Table 3:** Correlation between changes of plasma BDNF and factor scores of changes of cognitive functions and WHOQOL over 12 weeks in all BD patients and patients stratified by subtype of BD and *BDNF* Val66Met genotypes.

Correlation between changes of plasma BDNF levels and Composite factors of changes of cognitive function and WHOQOL Stratified by BD subtypes & BDNF Val66Met Polymorphism	Factor 1		Factor 2		Factor 3		Factor 4	
	(WCST)		(CPT impulsivity)		(CPT distractibility)		WHOQOL	
	*r*	*p*	*R*	*p*	*r*	*p*	*r*	*p*
All Patients	−0.25	0.00037	0.09	0.18	−0.07	0.30	−0.02	0.85
BP-I only								
*BDNF* Val66Met Polymorphism								
* *Val/Val genotype	0.10	0.78	−0.52	0.10	0.28	0.40	0.32	0.44
* *Val/Met genotype	−0.54	0.008	−0.13	0.54	−0.03	0.90	−0.39	0.15
* *Met/Met genotype	−0.37	0.21	0.59	0.03	−0.28	0.35	−0.13	0.69
BP-II only								
*BDNF* Val66Met Polymorphism								
* *Val/Val genotype	−0.27	0.09	−0.19	0.23	0.15	0.35	0.13	0.57
* *Val/Met genotype	−0.20	0.07	0.14	0.22	−0.01	0.96	0.03	0.84
* *Met/Met genotype	−0.08	0.64	0.21	0.19	−0.12	0.47	−0.19	0.40

^*^p < 0.05/24 = 0.002; **p < 0.001.
